# Supplementary material for: Dynamic swimming pattern of Pseudomonas aeruginosa near a vertical wall during initial attachment stages of biofilm formation
Source: Sci Rep. 2021 Jan 21;11:1952. doi: 10.1038/s41598-021-81621-w (PMC7820011; doi:10.1038/s41598-021-81621-w)
Supplement: Supplementary file 1 — Supplementary Information. [file 41598_2021_81621_MOESM1_ESM.docx]

**Supplementary Information**

**Title**

Dynamic swimming patterns of *Pseudomonas aeruginosa* near a vertical wall during initial attachment stages of biofilm formation.

**Author affiliation**

Nicole Zi-Jia Khong^1,*^, Yukai Zeng^2,*^,  Soak-Kuan Lai^1,*^, Cheng-Gee Koh^1^, Zhao-Xun Liang^1^, Keng-Hwee Chiam^2,#^, Hoi-Yeung Li^1,#^

*^1^ School of Biological Sciences, College of Science, Nanyang Technological University, Singapore*

*^2^ Bioinformatics Institute, A*STAR, Singapore*

*^*^ These authors contributed equally:*

*^#^ Corresponding author:*

*for light sheet imaging and experimental design -*[*hyli@ntu.edu.sg*](mailto:hyli@ntu.edu.sg)*,*

*for image and data analysis -*[*chiamkh@bii.a-star.edu.sg*](mailto:chiamkh@bii.a-star.edu.sg)

Figure S1. Preparation of incubation chamber. Materials: a 1ml tuberculin syringe (cut open at the tip), a 200μl yellow pipette tip (cut open at the tip) and a 2mm x 2mm x 70mm (WDL) styrene square rod. Step1: Wrap the bottom opening of the 1ml syringe tightly with parafilm. Step 2: Insert the styrene square rod into the cut yellow pipette tip. Step 3: Fill the 1ml syringe with 1% LB agarose. Step 4: Insert the styrene square rod into the syringe with LB agarose and let the agarose solidify. Position of the cuboid rod was stabilised in the syringe using the cut yellow pipette tip. Step 5: Cover the top opening of the syringe with parafilm loosely after the stained bacterial suspension was loaded into the chamber. Step 6. Release the chamber using the syringe plunger after it is mounted in the Zeiss light sheet Z1 microscope.

Figure S2. Near wall swimming behavior of *E. coli*. (A) Histograms of the speed of *E. coli*. (B) The turning angle, θ, for *E. coli* showed a broad distribution throughout. (C) *E. coli* speed vs. *h* which is the perpendicular distance to the wall. The red line is a least-squares fit to the form *v ~ h^-β^* for some exponent *β*, where *β*= 0.12 for *E. coli*. (D) *E. coli* shows right-handed turns only when it swam near a wall (E) The cell trajectory angle of approach towards the wall, φ, is obtained for each individual cell trajectory. The number of *E. coli* with φ ending to 0 increases as the distance to the wall *h* decreases.
